# Inflammation as well as angiogenesis may participate in the pathophysiology of brain radiation necrosis

**DOI:** 10.1093/jrr/rru017

**Published:** 2014-03-27

**Authors:** Erina Yoritsune, Motomasa Furuse, Hiroko Kuwabara, Tomo Miyata, Naosuke Nonoguchi, Shinji Kawabata, Hana Hayasaki, Toshihiko Kuroiwa, Koji Ono, Yuro Shibayama, Shin-Ichi Miyatake

**Affiliations:** 1 Department of Neurosurgery, Division of Life Sciences, Osaka Medical College, 2-7 Daigaku-machi, Takatsuki City, Osaka 569-8686, Japan; 2 Department of Pathology, Division of Life Sciences, Osaka Medical College, 2-7 Daigaku-machi, Takatsuki City, Osaka 569-8686, Japan; 3 Department of Anatomy and Cell Biology, Division of Life Sciences, Osaka Medical College, 2-7 Daigaku-machi, Takatsuki City, Osaka 569-8686, Japan; 4 Particle Radiation Oncology Research Center, Research Reactor Institute, Kyoto University, Japan

**Keywords:** brain radiation necrosis, CXCL12/CXCR4 chemokine axis, inflammation, microglia, pro-inflammatory cytokine

## Abstract

Radiation necrosis (RN) after intensive radiation therapy is a serious problem. Using human RN specimens, we recently proved that leaky angiogenesis is a major cause of brain edema in RN. In the present study, we investigated the same specimens to speculate on inflammation's effect on the pathophysiology of RN. Surgical specimens of symptomatic RN in the brain were retrospectively reviewed by histological and immunohistochemical analyses using hematoxylin and eosin (H&E) staining as well as immunohistochemical staining for VEGF, HIF-1α, CXCL12, CXCR4, GFAP, CD68, hGLUT5, CD45, IL-1α, IL-6 TNF-α and NF-*k*B. H&E staining demonstrated marked angiogenesis and cell infiltration in the perinecrotic area. The most prominent vasculature was identified as thin-walled leaky angiogenesis, i.e. telangiectasis surrounded by prominent interstitial edema. Two major cell phenotypes infiltrated the perinecrotic area: GFAP-positive reactive astrocytes and CD68/hGLUT5-positive cells (mainly microglias). Immunohistochemistry revealed that CD68/hGLUT5-positive cells and GFAP-positive cells expressed HIF-1α and VEGF, respectively. GFAP-positive cells expressed chemokine CXCL12, and CD68/hGLUT5-positive cells expressed receptor CXCR4. The CD68/hGLUT5-positive cells expressed pro-inflammatory cytokines IL-1α, IL-6 and TNF-α in the perinecrotic area. VEGF caused leaky angiogenesis followed by perilesional edema in RN. GFAP-positive cells expressing CXCL12 might attract CXCR4-expressing CD68/hGLUT5-positive cells into the perinecrotic area. These accumulated CD68/hGLUT5-positive cells expressing pro-inflammatory cytokines seemed to aggravate the RN edema. Both angiogenesis and inflammation might be caused by the regulation of HIF-1α, which is well known as a transactivator of VEGF and of the CXCL12/CXCR4 chemokine axis.

## INTRODUCTION

Radiotherapeutic technologies have progressed in recent decades; patients with malignant brain tumors can now be treated with high-dose irradiation with good conformity, prolonging their survival. On the other hand, brain radiation necrosis (RN), a late adverse effect of radiation therapies, has become a serious problem, and existing treatments for brain RN have not been sufficiently effective. Recently, bevacizumab (BV), an antibody to vascular endothelial growth factor (VEGF), has received attention as a promising treatment for RN [1–3]. BV shows sharp and potent treatment effects. On the basis of our analysis of human RN surgical specimens, we previously demonstrated that edema in RN is caused by VEGF overexpression in reactive astrocytes [4]. However, the effects are often temporary; RN occasionally recurs after BV treatments [1]. Therefore, to overcome this intractable pathology, it will be necessary to elucidate its underlying mechanisms.

**Figure 1.**
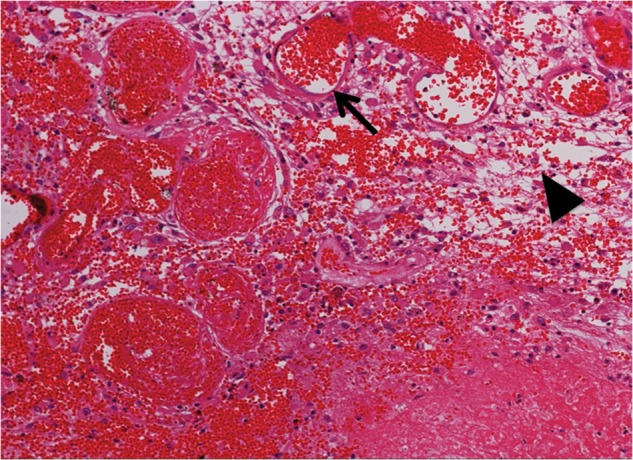
A hematoxylin and eosin (H&E)-stained specimen from Case 5. Thin-walled enlarged capillaries indicating telangiectasis (arrow) and the proliferation of arterioles can be seen in the area between the necrotic core and normal brain tissue. These blood vessels were accompanied by interstitial edema (arrowhead) due to plasma leakage. The original objective magnification was ×100.

In a recent study using human RN specimens, we proved that ‘leaky’ angiogenesis is a major cause of brain edema in RN [4], as shown in Fig. 1 in this report. Furthermore, we have discovered that GFAP-positive and CD68-positive cells accumulate around the circumference of the RN core, i.e. the perinecrotic area [5]. In addition, we have shown that HIF-1α and VEGF participate in the formation of angiogenesis, microbleeding, and interstitial edema at the RN circumference [4]. However, it remains to be determined why these GFAP-positive cells and CD68-positive cells accumulate in the perinecrotic area, which cells express VEGF and HIF-1α in RN, and whether or not other molecules participate in the pathophysiology of RN.

In malignant tumors (including glioblastomas) the CXCL12/CXCR4 chemokine axis is known to be correlated with HIF-1α and VEGF expression [6, 7]. Furthermore, the involvement of chronic inflammation has also been suggested as a mechanism (in addition to angiogenesis) underlying brain RN [8]. The aim of the present study is to elucidate the molecular mechanisms underlying brain RN in humans, with special reference to angiogenesis and inflammation. We also speculated on the potential relationship between chemokine and cytokine expression and accumulation in GFAP, CD68, hGLUT5 and CD45-positive cells in the perinecrotic area. Here, GFAP, CD68, hGLUT5 and CD45 were adopted as markers for astrocytes, monocytes, microglias and lymphocytes, respectively. The study design consisted of a retrospective qualitative review with histological and immunohistochemical analyses of surgical specimens from the brains of patients with symptomatic RN treated in our department.

## MATERIALS AND METHODS

### Specimens

Surgical specimens of seven patients with symptomatic RN in the brain who had been treated in our department from 2006 through 2009 were subjected to histological and immunohistochemical analyses. The study was approved by the Osaka Medical College Ethics Committee, and we obtained informed consent from all of the patients prior to surgeries and procedures. All of the patients had received radiation therapy, including X-ray treatment, stereotactic radiosurgery (SRS), and/or boron neutron capture therapy [9–13]. They also had received some medical treatments for RN, including corticosteroids, anticoagulants, and so on, for at least one month; however, the symptoms were refractory to these medical treatments. The original diseases were two head and neck cancers, four glioblastomas, and one metastatic brain tumor derived from breast cancer. Both patients with head and neck cancers had only extracranial tumors without infiltration and/or metastasis to the brain. Their irradiation fields included not only the original head and neck cancers but also some part of the temporal lobe. Therefore, these were samples of pure RN without the presence of tumor cells in the temporal lobe. Brain RN in all cases was diagnosed by pathological examination of the surgical specimen (Table 1).

**Table 1.** Patient profile of symptomatic radiation necrosis

Case	Age	Gender	Original dis.**^a^**	Radiation**^b^**	Duration**^c^**	Chemotherapy^d^
1	78	M	Sal. Duc. Ca.	XRT (60 Gy), BNCT × 2 (6.1 and 10.1 Gy-Eq)	20	
2	46	F	Ade. Ca.	XRT (60 Gy), BNCT × 2 (6.9 and 6.7 Gy-Eq)	7	MTX, UFT, CDDP
3	69	F	GB	XRT (60 Gy), BNCT (13.3 Gy-Eq)	3	ACNU
4	68	F	GB	XRT (60 Gy), BNCT (9.67 Gy-Eq)	12	
5	46	M	GB	XRT (60 Gy), BNCT (13.7 Gy-Eq)	5	PCV
6	39	M	GB	XRT (60 Gy), SRS (18 Gy)	8	ACNU
7	54	F	Ade. Ca.	SRS (22 Gy)	9	Herceptin

**^a^**Sal. Duc. Ca. = salivary ductal carcinoma, GB = glioblastoma, Ade. Ca. = adenocarcinoma. **^b^**XRT = X-ray treatment, BNCT = boron neutron capture therapy, SRS = stereotactic radiosurgery, Gy-Eq = biologically equivalent X-ray dose that would have equivalent effects on tumor and normal brain. In BNCT, the presented dose is the peak point dose for normal brain. In SRS, the presented dose is the marginal X-ray or gamma-ray dose. BNCT × 2 means intentional fractionated BNCT in two sessions. **^c^**Months between termination of last radiotherapy and onset of symptoms caused by radiation necrosis. Radiation necrosis in Cases 1 and 2 occurred in temporal lobes and was included in the irradiation fields. ^d^MTX = methotrexate, UFT = combined drug of tegafur and uracil, CDDP = cisplatin, ACNU = nimustin, PCV = procarbazine, nimustin, vincristine.

### Tissue preparation and immunohistochemistry

Tissue samples were fixed in 10% buffered formalin and embedded in paraffin. Tissue sections were cut (4-μm slices), deparaffinized with xylene, and rehydrated through graduated ethanol solutions. One section of each sample was stained with hematoxylin and eosin (H&E), and the other sections were used for HIF-1α, VEGF, CXCL12, CXCR4, IL-1α, IL-6, TNF-α, GFAP, CD68, hGLUT5, CD45 and NF-*κ*B staining by the biotin–streptavidin–peroxidase method. Here, GFAP, CD68, hGLUT5 and CD45 were adopted as markers for astrocytes, monocytes, microglias and lymphocytes, respectively, as described above. Deparaffinized and rehydrated sections were subjected to pressure boiling for antigen retrieval in antigen unmasking solution. These sections were incubated with 0.03% hydrogen peroxidase for 5 min at room temperature to block endogenous peroxidase activity. The sections were then incubated with one of the above 12 primary antibodies at 4°C overnight. The information on these primary antibodies, including dilutions, is summarized in Table s1. The sections were then incubated with peroxidase-labeled secondary antibody (Dako) for 30 min at room temperature. They were developed with diaminobenzidine, lightly counterstained with hematoxylin, and mounted.

### Double immunofluorescence microscopy

Double immunofluorescence staining was performed using the following antibody combinations: CXCL12 and GFAP or CD68, CXCR4 and GFAP or CD68, HIF-1α and GFAP or CD68, VEGF and GFAP or CD68, IL-1α and GFAP or CD68, IL-6 and GFAP or CD68, TNF-α and GFAP or CD68, hGLUT5 and CD68 or CXCR4 or IL-1α or HIF-1α, and CD45 and IL-1α or CXCR4. The secondary antibodies used were Alexa Fluor 488 goat anti-mouse IgG, Alexa Fluor 488 goat anti-rabbit IgG, Alexa Fluor 546 goat anti-mouse IgG, and Alexa Fluor 546 goat anti-rabbit IgG (Invitrogen, Carlsbad, CA). These were examined using an LSM510 laser scanning confocal microscope (Carl Zeiss, Oberkochen, Germany).

## RESULTS

### Histochemical analysis with H&E staining

Figure 1 shows a typical finding of H&E staining of RN from Case 5. Remarkable angiogenesis existed in the perinecrotic area. The most prominent vasculature was identified as a thin-walled, leaky angiogenesis, such as telangiectasis with prominent interstitial edema, which is consistent with our previous study [4]. The interstitial edema was probably caused by leakage of the plasma from the fragile angiogenesis. There was no cytoarchitecture in the core of the necrotic foci, whereas some infiltrating cells were observed in the perinecrotic area, as indicated with hematoxylin-stained nuclei. Of course, the incidence of RN is generally affected by the applied radiation dose, distribution, and adjuvant chemotherapy [14, 15]. However, these are universal pathological findings, irrespective of the original tumor types and radiation modalities, as we reported previously [4].

### Immunohistochemical analysis of the localization of CXCL12, CXCR4, GFAP, CD68, IL-1α, IL-6, TNF-α, NF-κB, hGLUT5 and CD45

**Figure 2.**
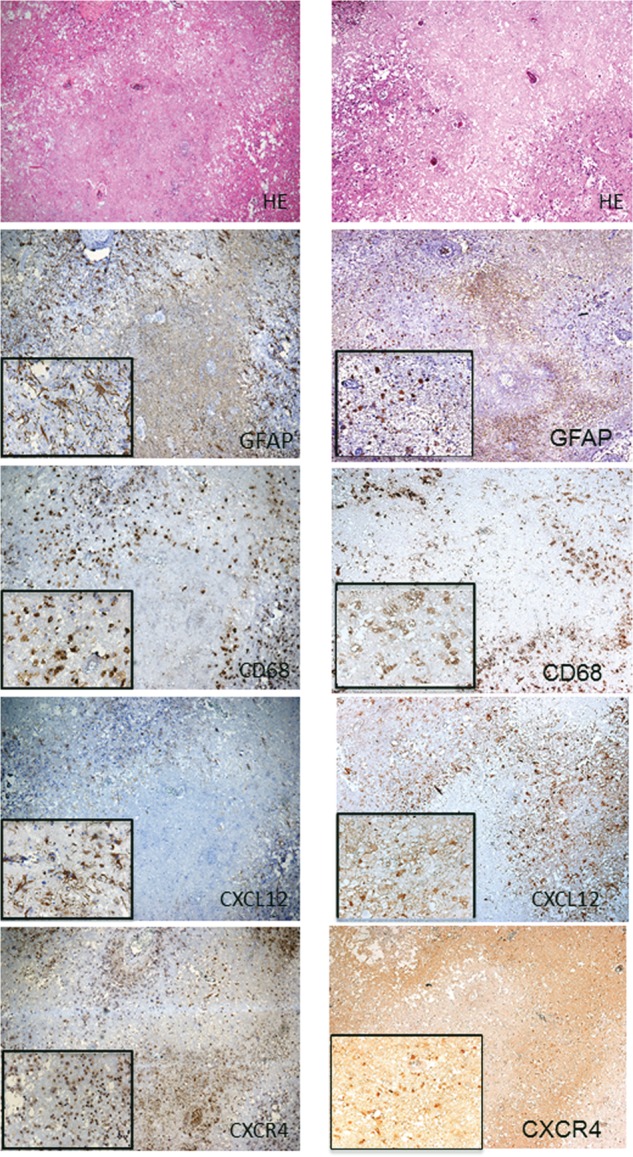
H&E staining and immunohistochemistry for GFAP, CD68, CXCL12 and CXCR4 of the surgical specimen from Case 2 in the left column and for Case 3 in the right column. H&E staining showed the necrotic core and the surrounding tissue (perinecrotic area). Immunohistochemical staining for GFAP, CD68, CXCL12 and CXCR4 revealed GFAP- and CXCL12-positive astrocytic cells, and CD68- and CXCR4-positive oval cells in the perinecrotic area. CD68- and CXCR4-positive cells were also observed in small numbers inside the necrotic core. On the other hand, no or scarce immunoreactivity of GFAP and CXCL12 was observed in the necrotic core. The original objective magnifications were ×40 and ×200.

We next sought to determine the cell types (i.e. astrocytes or monocytes) that produce chemokines and/or express the corresponding receptors. To this end, we performed enzyme-immunohistochemical analyses for gross identification of the infiltrative cells and proteins expressed using the primary antibodies described in Materials and Methods. H&E staining was also performed on each specimen to identify the necrotic core, the perinecrotic area, and the normal brain area. The specimens obtained from Cases 2 and 3 demonstrate representative findings for the expression of GFAP, CD68, CXCL12 and CXCR4 (Fig. 2). As we reported previously [5], two major cell phenotypes infiltrated the perinecrotic area: GFAP-positive astrocytes and CD68-positive monocytes. We observed similar distributions of GFAP-positive cells and CXCL12-positive cells; CD68-positive cells and CXCR4-positive cells were also similarly distributed. The former cell type was limited mainly to the perinecrotic area, whereas the latter population was observed not only in the perinecrotic area but also, though to a lesser degree, in the necrotic core. The same tendencies were observed among all cases (data not shown).

To evaluate the distribution of cytokine production in RN, we performed immunohistochemical staining for IL-1α, IL-6 and TNF-α. The specimens obtained from all cases revealed oval cells positive for IL-1α, IL-6 and TNF-α in the perinecrotic area (data not shown). These cells also appeared inside the necrotic core, though to a lesser degree. We then examined the expression of NF-*κ*B as a master molecule of inflammation in RN using the Case 2 specimens. Interestingly, NF-*κ*B was expressed not in normal brain tissue but in the perinecrotic area (Fig. *s*1). This molecule was expressed not only in the cytoplasm but also in the nucleus, and was morphologically assessed as a monocyte. The same tendency was confirmed in Case 3.

Subsequently, we focused on immune cells in the brain to determine their effect on RN. To this end, we performed an immunohistochemical study using hGLUT5, CD68 and CD45 antibodies, as stated above. We observed similar distributions in hGLUT5-positive cells and CD68-positive cells in Cases 3 and 4 (Fig. *s2*). However, the number of CD45-positive cells was limited; their distribution pattern was distinguishable from those of GFAP-positive cells and CD68- and hGLUT5-positive cells. The same tendencies were observed in Cases 6 and 7 (data not shown).

Taken together, these data suggested that CXCL12 and CXCR4 might be expressed in GFAP-positive reactive astrocytes and hGLUT5- and/or CD68-positive cells, respectively. Also, microglias, macrophages, and even lymphocytes, each of which can produce proinflammatory cytokines, accumulated in the perinecrotic area. Therefore, inflammation might play a significant role in the pathogenesis of RN in the brain.

### Relationships between CXCL12, CXCR4, HIF-1α, VEGF, IL-1α, IL-6 and TNF-α expression and GFAP/CD68 expression in radiation necrosis

**Figure 3.**
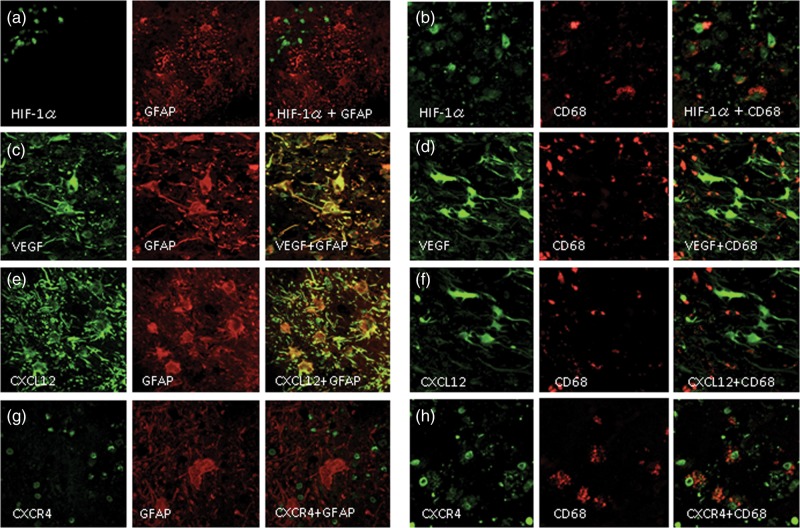
Double immunofluorescence staining of the specimen from Case 2. The expression of HIF-1α was not detected in reactive astrocytes, as revealed by GFAP (**a**), but was detected in CD68-positive cells (**b**). VEGF was expressed in GFAP-positive cells (**c**) but was hardly expressed in CD68-positive cells (**d**). Similarly, CXCL12 was expressed in GFAP-positive reactive astrocytes (**e**) but not in CD68-positive cells (**f**). In contrast, CXCR4 was not expressed in GFAP-positive cells (**g**) but was expressed in CD68-positive cells (**h**). The original objective magnification was ×400.

**Figure 4.**
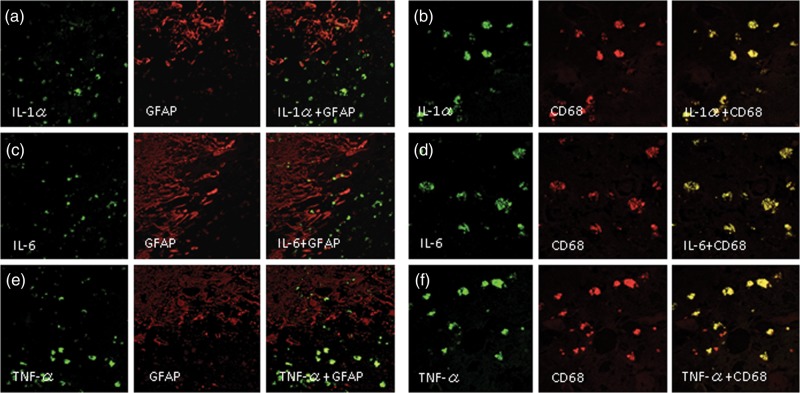
Double immunofluorescence staining of Case 2. IL-1α, IL-6 and TNF-α were not expressed in reactive astrocytes, as revealed by GFAP-positive cells (**a**, **c**, **e**), but were expressed in CD68-positive cells (**b**, **d**, **f**). The original objective magnification was ×400.

In order to further examine the distribution of cells responsible for the expression of cytokines and chemokines, we performed double immunofluorescence staining. The specimen from Case 2 provided a representative pattern (Figs 3 and 4). Figure 3 shows that the expression of HIF-1α was recognized not in GFAP-positive cells (a) but in most CD68-positive cells (b). However, some CD68-positive cells did not produce HIF-1α, while some CD68-negative cells did. VEGF was expressed in GFAP-positive cells (c) but was hardly expressed in CD68-positive cells (d). Likewise, the expression of CXCL12 was detected in GFAP-positive cells (e) but not in CD68-positive cells (f). In contrast, CXCR4 was not expressed in GFAP-positive cells (g) but was expressed in CD68-positive cells (h). However, some CXCR4 expression was recognized in CD68-negative cells.

Furthermore, Fig. 4 shows that IL-1α, IL-6 and TNF-α were not expressed in GFAP-positive cells (a, c, e) but were expressed in CD68-positive cells (b, d, f). The same tendencies were confirmed in the other specimens (data not shown).

### Relationships between CXCR4, HIF-1α and IL-1α expression and hGLUT5/CD68 and CD45 expression in radiation necrosis

In order to identify the cells that are responsible for inflammation in RN, we analyzed double immunofluorescence using anti-CD68, anti-hGLUT5 and anti-CD45 antibodies. We used the former two antibodies to distinguish microglias from macrophages originating from peripheral blood in CD68-positive cells. Figure *s3* (Case 1) shows that CXCR4, HIF-1α and IL-1α were expressed in many hGLUT5-positive cells (a, b, c). However, some CXCR4, HIF-1α and IL-1α-positive cells did not express hGLUT5 and vice versa. Also, most CD68-positive cells were identical to hGLUT5-positive cells, although some cells expressed only CD68 or hGLUT5 (d). Almost no CD45-positive cells produced IL-1α. Interestingly, almost all CD45-positive cells co-expressed CXCR4 (data not shown). This is confirmed in the other cases.

## DISCUSSION

Medical treatment with the anti-VEGF antibody BV, or surgical resection of necrotic tissue containing VEGF-producing cells may serve to decrease perilesional edema and immediately improve symptoms stemming from the efficient reduction of VEGF in the perinecrotic area [1–3, 5]. These phenomena suggest that VEGF is the key molecule in the pathogenesis of RN. Here the role of reactive astrocytes in RN is clearly the production of VEGF, while that of monocytes remains unclear. The present results prove that these monocytes in the perinecrotic area produce HIF-1α. HIF-1α is a well-known transactivator not only for VEGF, but also for the CXCL12-CXCR4 axis [6, 7, 16]. The CXCL12-CXCR4 axis is also well known to be a chemotactic factor and to play a significant role in inflammation. Therefore, we examined the expression of these molecules in RN. As we had speculated, CXCR4-positive monocytes and lymphocytes gathered in the perinecrotic area.

Yoshii *et al.* stressed the mechanism underlying the development and progress of late cerebral RN, with special emphasis on inflammatory responses [8]. They put forward the following hypothesis to explain the mechanism underlying RN. First, irradiation damages endothelial cells, causing the blood–brain barrier (BBB) to fail. The inflammatory cells then cross into the extravascular space. At the same time, these cells secrete cytokines that cause other inflammatory cells to develop. This effect becomes an uncontrolled inflammatory response and continues, becoming chronic inflammation [8]. Thereafter we examined the role of inflammation in RN more precisely by using immunohistochemistry and double immunofluorescence. Kureshi *et al.* reported that macrophages and some astrocytes might migrate to the RN area and produce IL-1α, TNF-α and IL-6 [17]; this would be partly consistent with our present results, although the exact cell types corresponding to each type of cytokine production were ambiguous in their report. In laboratory animal RN models, a key cytokine is TNF-α, which regulates other pro-inflammatory cytokines to increase BBB permeability and leukocyte adhesion, to activate astrocytes, and to induce endothelial apoptosis [18–21]. Moreover, IL-1α, either alone or in cooperation with TNF-α and IL-6 in the downstream region, exacerbates inflammation [22].

The roles of chemokine CXCL12 and its receptor CXCR4 have been well established in the immune and nervous systems, where they localize in various cell types with specific microenvironments [23]. CXCR4 is expressed not only in monocytes, but also in lymphocytes [24, 25]. In the present study, CD45-positive cells expressed CXCR4, as described in Results. Thereafter, these lymphocytes might be drawn into the perinecrotic area from peripheral blood by homing; however, unlike CD68- and/or hGLUT5-positive cells, they did not produce pro-inflammatory cytokines. At first, in this study, we used CD68 antibody to detect monocytes. But it was difficult for us to discriminate microglia from a macrophage in brain tissue using only this marker. Horikoshi and Sasaki *et al.* reported that the hGLUT5 antibody is a good and specific marker for microglia [26–32]. We therefore used it to recognize microglia. In the present study, CD68 and hGLUT5 were usually expressed in many identical cells, although some hGLUT5-positive cells did not show CD68 expression and vice versa. HIF-1α, CXCR4 and IL-1α expression was confined largely to hGLUT5-positive cells, but some of them were expressed in CD68-positive and hGLUT-5–negative cells, as stated in Results. Also, as described above, CD45 cells in the perinecrotic area expressed CXCR4 but not inflammatory cytokine. Taken together, these findings suggest that microglia play a key role in this inflammation, but some macrophages from peripheral blood may also be involved. However, lymphocytes do not participate in the production of pro-inflammatory cytokines.

We also examined NF-*κ*B expression in the perinecrotic area (Fig. *s*1). At a glance, this molecule was expressed in monocytes in the perinecrotic area. NF-*κ*B is well known as a major molecule of inflammation. As the function of NF-*κ*B in RN is unclear, further examination is needed to elucidate this molecule's function. In culture conditions, CXCR4-positive microglia can produce IL-6 with CXCL12 stimuli via an NF-*κ*B-dependent pathway [33]. This may suggest a role of NF-*κ*B in RN. Also, it is observed in cerebral ischemia that CXCL12 is upregulated under ischemic conditions, thereby inducing monocytes to gather in the ischemic penumbra and a subsequent inflammatory response [34–36]. These observations support chemokine–cytokine interaction in RN.

**Figure 5.**
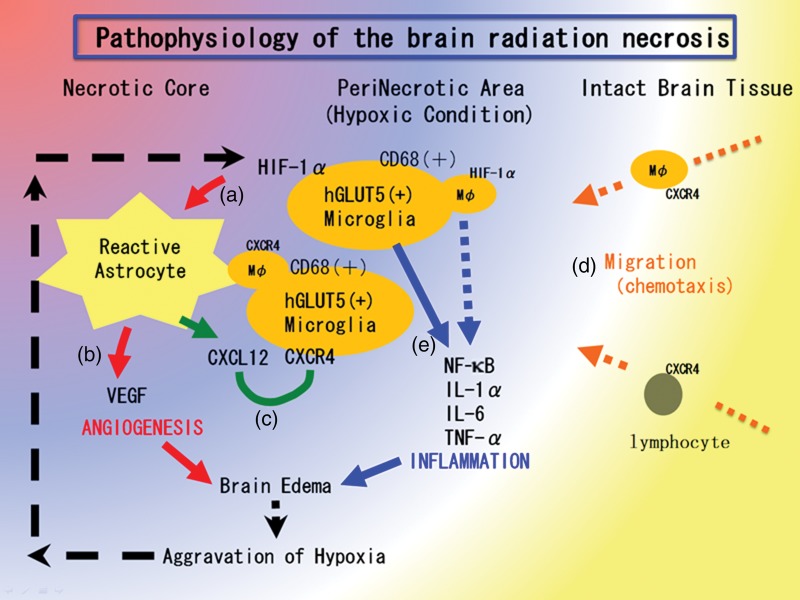
Hypothesis of the pathophysiology of brain radiation necrosis. (**a**) Vascular damage around the irradiated tumor tissue caused tissue ischemia. This hypoxia induced hGLUT5-positive microglias to express HIF-1α around the necrotic core. (**b**) Under HIF-1α regulation, VEGF was expressed in reactive astrocytes, causing leaky and fragile angiogenesis. (**c**) CXCL12/CXCR4 signaling is also regulated by HIF-1α. (**d**) CXCL12-expressing reactive astrocytes might draw CXCR4-expressing macrophages and lymphocytes by chemotaxis into the perinecrotic area. (**e**) These accumulated hGLUT5-positive microglias producing NF-κβ and pro-inflammatory cytokines seemed to aggravate radiation necrosis.

From the observed qualitative data in this study, let us summarize our original hypothesis about RN pathophysiology (Fig. 5). That is, the first step in a brain undergoing radiation therapy and developing necrosis is blood vessel damage just around the tumor. This is connected with hypoxia close to the irradiated tumor tissue, which causes the upregulation of HIF-1α in hGLUT5-positive microglias. Because HIF-1α is well known as a transactivator of VEGF and CXCL12/CXCR4 signaling [7, 16], the upregulation of HIF-1α augments VEGF and CXCL12 expression in GFAP-positive reactive astrocytes. The former produced the leaky and fragile angiogenesis and the subsequent perilesional edema in RN. The latter might draw CXCR4-expressing hGLUT5-positive microglias and CXCR4-expressing lymphocytes by chemotaxis to the perinecrotic area. The production of pro-inflammatory cytokines by these accumulated hGLUT5-positive cells seemed to aggravate the perilesional edema. However, although some CD45-positive lymphocytes gathered in the perinecrotic area, they were not involved in pro-inflammatory cytokine production. NF-*κ*B must play a significant role in RN inflammation. The aggravation of edema could lead to the further development of focal ischemia, which augments the expression of HIF-1α in the microglias in the perinecrotic area. Here, both angiogenesis and inflammation may contribute to a synergistic and malignant RN cycle. These hypotheses need to be confirmed in experimental animal models, as described below. In any case, the present results suggest that inflammation participates in the pathophysiology of brain RN.

The present research appears to have elucidated a part of the molecular mechanism underlying brain RN on the basis of qualitative histology and immunohistochemistry. In order to prove these hypotheses more conclusively, it will be necessary to create a brain RN model in laboratory animals and to analyze the pathophysiology in each stage. An improved understanding of the mechanism by which the numerous cytokines in brain RN are regulated could play an important role in the formulation of treatment strategies. If a brain RN becomes controllable, it will further advance the application of radiation therapy to central nervous system tumors, leading to the conquest of intractable malignant brain tumors. Furthermore, the quality of life of patients would certainly be improved by an effective treatment for symptomatic RN of the brain.

## SUPPLEMENTARY DATA

Three supplementary figures (Figs *s*1, *s2* and *s3*), one supplementary table (Table *s*1), and an appendix are available at the *Journal of Radiation Research* online.

## FUNDING

Funding to pay Open Access publication charges for this article was provided by a Grant-in-Aid for Scientific Research (B) (23390355) and by a Grant-in-Aid for Exploratory Research (24659658) to S-I.M., and by a Grant-in-Aid for Scientific Research (C) (23592145) to M.F. from the Japanese Ministry of Education, Culture, Sports, Science, and Technology.

## Supplementary Material

Supplementary Data
