# Supplementary material for: Inflammation as well as angiogenesis may participate in the pathophysiology of brain radiation necrosis
Source: J Radiat Res. 2014 Mar 27;55(4):803–11. doi: 10.1093/jrr/rru017 (PMC4100008; doi:10.1093/jrr/rru017)
Supplement: Supplementary Data [file supp_55_4_803__index.html]

Inflammation as well as angiogenesis may participate in the pathophysiology of brain radiation necrosis — Inflammation as well as angiogenesis may participate in the pathophysiology of brain radiation necrosis — Supplementary Data 

# Inflammation as well as angiogenesis may participate in the pathophysiology of brain radiation necrosis

## Supplementary Data

Supplementary Data

**Files in this Data Supplement:**

- Supplementary Data - Doc file
- Supplementary Figure 1 - tif file
- Supplementary Figure 2 - tif file
- Supplementary Figure 3 - tif file
- Supplementary Table 1 - docx file
