# Supplementary material for: Inflammation as well as angiogenesis may participate in the pathophysiology of brain radiation necrosis
Source: J Radiat Res. 2014 Mar 27;55(4):803–11. doi: 10.1093/jrr/rru017 (PMC4100008; doi:10.1093/jrr/rru017)
Supplement: Supplementary Data [file supp_rru017_rru017supp.doc]

Appendix

***Figure legends for supplementary figures 1***

Supplementary Fig. 1. Immunohistochemistry for NF-*κ*B in case 2. NF-*κ*B was recognized in the perinecrotic area (a and b). The molecule was expressed in the cytoplasm and the nucleus in monocyte-like cells. But no immunoreactivity of NF-*κ*B was observed in normal brain tissue in the same specimen. The original objective magnification was x200.

Fig. *s2*.　 H&E staining and immunohistochemistry for hGLUT5, CD68, and CD45 of the surgical specimen from case 3 in the left column and case 4 in the right column. Immunohistochemical staining for hGLUT5, CD68, and CD45 showing hGLUT5-, CD68-, and CD45-positive cells in the perinecrotic area. The distributions of hGLUT5-positive cells and CD68-positive cells were similar. However, CD45-positive cells expressed at low levels and were similar to neither GFAP-positive cells nor CD68-positive cells. The original objective magnifications were x40 and x200.

Fig. *s2*. Double immunofluorescence staining in case 1. CXCR4, HIF-1α, and IL-1α were expressed in hGLUT5-positive cells (a, b, c). On the other hand, a portion of the CD68-positive cells corresponded to hGLUT5-positive cells, and CD68-positive, hGLUT5-negative cells were present (d). The original objective magnification was x400.
