# Supplementary figures and images for: Inflammation as well as angiogenesis may participate in the pathophysiology of brain radiation necrosis
Source: J Radiat Res. 2014 Mar 27;55(4):803–11. doi: 10.1093/jrr/rru017 (PMC4100008; doi:10.1093/jrr/rru017)

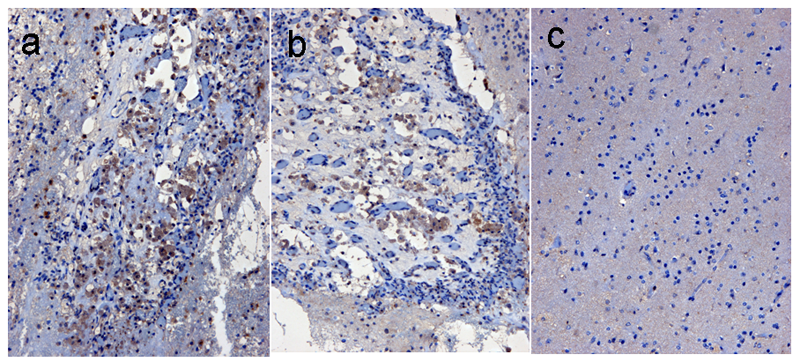

Supplement: Supplementary Data [file supp_rru017_rru017supp_fig1.tif]

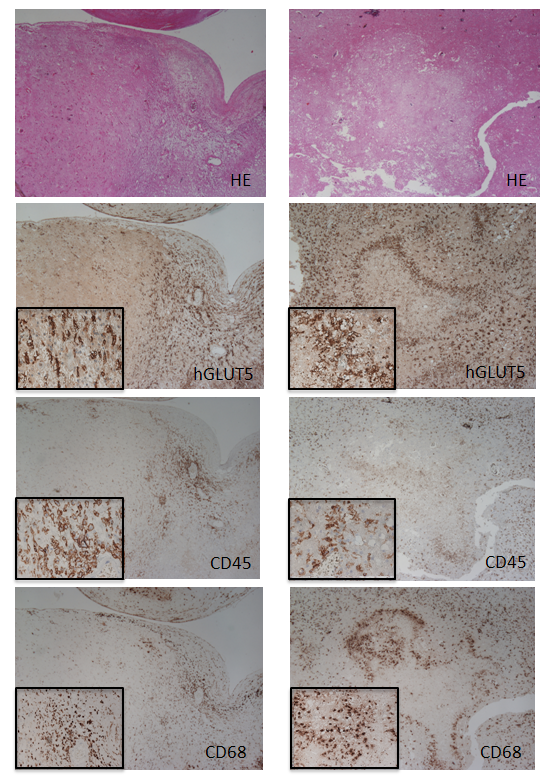

Supplement: Supplementary Data [file supp_rru017_rru017supp_fig2.tif]

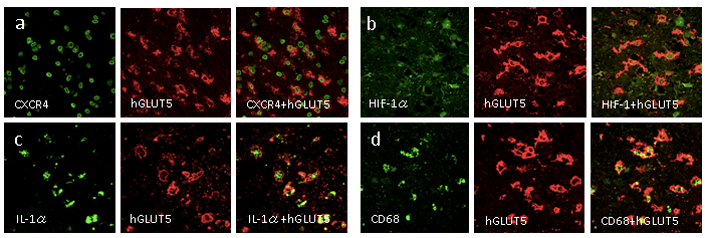

Supplement: Supplementary Data [file supp_rru017_rru017supp_fig3.tif]
