# Supplementary material for: Inflammation as well as angiogenesis may participate in the pathophysiology of brain radiation necrosis
Source: J Radiat Res. 2014 Mar 27;55(4):803–11. doi: 10.1093/jrr/rru017 (PMC4100008; doi:10.1093/jrr/rru017)
Supplement: Supplementary Data [file supp_rru017_rru017supp_table1.docx]

Supplementary Table １ 　Primary antibodies used for immunohistochemistry

| **Antibody** | **Clone** | **Company** | **Species** | **Pretreatment** | **Dilution** |
| --- | --- | --- | --- | --- | --- |
| HIF-1α | Polyclonal | Santa Cruz Biotechnology, Santa Cruz, CA | Rabbit | Target Retrieval Solution pH9 | 1:50 |
| VEGF | EP1176Y | Epitomics, Burlingame, CA | Rabbit | Target Retrieval Solution pH9 | 1:50 |
| CXCL12 | 79018 | R&D Systems, Minneapolis, MN | Mouse | Target Retrieval Solution pH9 | 1:20 |
| CXCR4 | 44716 | R&D Systems, Minneapolis, MN | Mouse | Target Retrieval Solution pH9 | 1:20 |
| IL-1α | 4414 | R&D Systems, Minneapolis, MN | Mouse | Target Retrieval Solution pH9 | 1:20 |
| IL-6 | 1936 | R&D Systems, Minneapolis, MN | Mouse | Target Retrieval Solution pH9 | 1:20 |
| TNF-α | 28401 | R&D Systems, Minneapolis, MN | Mouse | Target Retrieval Solution pH9 | 1:20 |
| hGLUT5 | OH-518 | IBL, Tokyo, Japan | Rabbit | Target Retrieval Solution pH9 | 1:200 |
| CD45 | EP322Y | Epitomics, Burlingame, CA | Rabbit | Target Retrieval Solution pH9 | 1:200 |
| GFAP | 6F2  Polyclonal | Dako, Glostrup, Denmark  Dako, Glostrup, Denmark | Mouse  Rabbit | Target Retrieval Solution pH9  Target Retrieval Solution pH9 | 1:50  1:500 |
| CD68 | KP1  EPR1392Y | Dako, Glostrup, Denmark  Epitomics, Burlingame, CA | Mouse  Rabbit | Target Retrieval Solution pH9  Target Retrieval Solution pH9 | 1:50  1:50 |
| NFkB | p65 | Abcam, Cambridge, MA, USA | Rabbit | Target Retrieval Solution pH9 | 1:50 |
